# Supplementary material for: Accuracy of point-of-care SARS-CoV-2 detection using buccal swabs in pediatric emergency departments
Source: Microbiol Spectr. 2024 Oct 29;12(12):e01884-24. doi: 10.1128/spectrum.01884-24 (PMC11619445; doi:10.1128/spectrum.01884-24)
Supplement: Supplemental tables and figures — Tables S1 and S2; Fig. S1 and S2. [file spectrum.01884-24-s0001.docx]

**Online-Only Supplement**

**eTable 1.** This table provides patient-level description of all participants who tested positive on the ID NOW™ buccal swab but negative on the nasopharyngeal RT-PCR.

**eTable 2.** Logistic regression model with the dependent variable being test agreement (i.e., same result from nasopharyngeal swab-RT-PCR test and buccal swab-ABBOTT ID NOW^™^ test).

**eFigure 1.** Cycle threshold (Ct) density curve depicting values for concordant and discordant swabs.

**eFigure 2.** Faces Pain Scale Revised Questionnaire scores assigned by participants 5 - < 12 years.

**eTable 1.** This table provides patient-level description of all participants who tested positive on the ID NOW™ buccal swab but negative on the nasopharyngeal RT-PCR.

| **Age (years)** | **Sex** | **Chronic Underlying Condition** | **# of COVID Vaccination Doses Received** | **Close Contact with a Probably/Confirmed SARS-CoV-2 Case** | **Symptoms** | **Symptom Duration** |
| --- | --- | --- | --- | --- | --- | --- |
| 0.0 | Male | No | Unvaccinated | Yes | Fever, Cough | 1 day |
| 0.1 | Male | No | Unvaccinated | No | Fever | < 1 day |
| 0.1 | Male | No | Unvaccinated | No | Cough, Shortness of breath, Vomiting | > 7 days |
| 0.1 | Male | No | Unvaccinated | Yes | Fever, Cough, Congestion | < 1 day |
| 0.2 | Female | No | Unvaccinated | Yes | Sputum, Fever, Cough, Congestion, Abdominal pain, Vomiting | < 1 day |
| 0.2 | Male | Laryngomalacia | Unvaccinated | Yes | Cough, Shortness of breath | >7 days |
| 0.3 | Male | No | Unvaccinated | No | Sputum, Fever, Cough, Congestion | 3 days |
| 0.5 | Male | No | Unvaccinated | Yes | Fever, Congestion | 2 days |
| 0.9 | Female | No | Unvaccinated | No | Fever, Congestion | 3 days |
| 1.0 | Male | No | Unvaccinated | No | Fever, Cough, Shortness of breath, Vomiting | 5 days |
| 2.0 | Female | Leukodystrophy, Dystopia | Unvaccinated | No | Fever, Shortness of breath, Wheezing, Sore throat, Vomiting | 2 days |
| 3.0 | Male | No | Unvaccinated | No | Sputum, Fever, Cough, Congestion, Abdominal pain, Vomiting | 4 days |
| 3.0 | Male | No | Unvaccinated | No | Fever, Cough, Congestion | 2 days |
| 4.0 | Male | Developmental delay | Unvaccinated | No | Fever, Cough, Congestion, Shortness of breath, Vomiting | > 7 days |
| 7.0 | Female | No | Unvaccinated | No | Fever, Headache, Cough, Abdominal pain, Vomiting | < 1 day |

**eTable 2. Logistic regression model with the dependent variable being test agreement (i.e., same result from nasopharyngeal swab-RT-PCR test and buccal swab-ABBOTT ID NOW^™^ test).**

| **Characteristic** | **Adjusted Odds Ratio** | **95% Confidence Interval** |
| --- | --- | --- |
| **Female** | 0.94 | 0.68, 1.29 |
| **Age** | 1.06 | 0.72, 1.58 |
| **Buccal swab performer** | | |
| Study team member | — | — |
| Participant | 1.06 | 0.57, 2.00 |
| Caregiver | 0.96 | 0.58, 1.59 |
| **Vaccination status** | | |
| Unvaccinated | — | — |
| 1 dose | 0.58 | 0.31, 1.08 |
| ≥2 doses | 1.01 | 0.60, 1.69 |
| **Duration of symptoms** | | |
| Asymptomatic | — | — |
| < 1 day | 1.17 | 0.14, 9.95 |
| 1 day | 2.09 | 0.24, 18.3 |
| 2 days | 1.12 | 0.13, 9.61 |
| 3 days | 2.10 | 0.24, 18.3 |
| 4 days | 2.26 | 0.25, 20.3 |
| 5 days | 2.37 | 0.26, 21.4 |
| 6+ days | 2.32 | 0.27, 19.7 |
| **Site** | | |
| Smaller sites* | — | — |
| Alberta Children's Hospital | 0.92 | 0.36, 2.35 |
| British Columbia Children's Hospital | 1.06 | 0.40, 2.80 |
| Centre Hospitalier de l'Universite de Laval | 1.80 | 0.61, 5.36 |
| Children's Hospital of Eastern Ontario | 1.17 | 0.42, 3.23 |
| Centre Hospitalier Universitaire Ste-Justine | 0.63 | 0.22, 1.82 |
| Izaak Walter Killam Health Centre | 7.88 | 0.91, 68.4 |
| McMaster Children's Hospital | 3.84 | 1.27, 11.6 |
| Montreal Children's Hospital | 1.42 | 0.43, 4.67 |
| Stollery Children's Hospital | 1.11 | 0.41, 2.99 |
| The Hospital for Sick Children | 1.46 | 0.56, 3.83 |

***** Kingston, Pattison, Janeway, London all had < 40 participants. Those four sites were combined into a single one called ‘Smaller sites’.

**eFigure 1. Cycle threshold (Ct) density curve depicting values for concordant (grey) and discordant (yellow) swabs. The density curve is a graphical representation of the relative frequency distribution of a continuous variable (Ct) with the curve enclosing an area of 1 above the x-axis. Values available for 273 participants.**


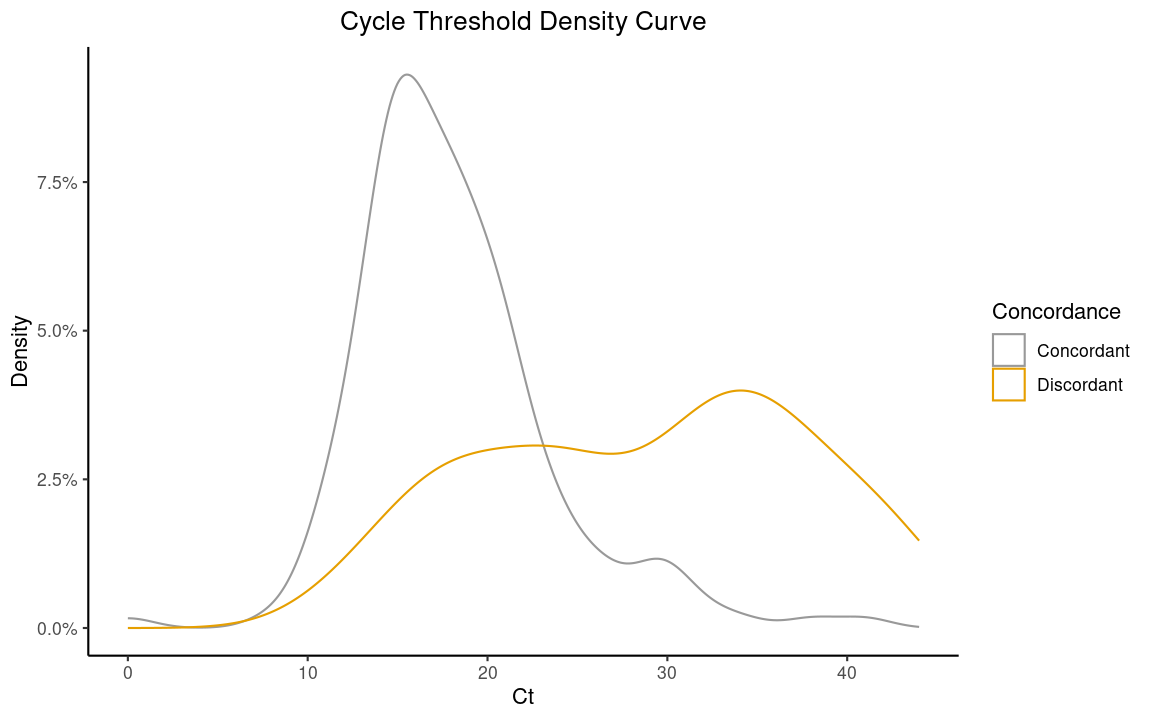


**eFigure 2. Faces Pain Scale Revised Questionnaire scores assigned by participants 5 - < 12 years. Scores were provided related to buccal swab by 586, and nasopharyngeal swab by 584 participants. A score of 0 indicates ‘no pain’ while 10 is ‘the worst pain’.**
